# Interactive metagenomic visualization in a Web browser

**DOI:** 10.1186/1471-2105-12-385

**Published:** 2011-09-30

**Authors:** Brian D Ondov, Nicholas H Bergman, Adam M Phillippy

**Affiliations:** 1 National Biodefense Analysis and Countermeasures Center, 110 Thomas Johnson Drive, Frederick, MD 21702, USA

## Abstract

**Background:**

A critical output of metagenomic studies is the estimation of abundances of taxonomical or functional groups. The inherent uncertainty in assignments to these groups makes it important to consider both their hierarchical contexts and their prediction confidence. The current tools for visualizing metagenomic data, however, omit or distort quantitative hierarchical relationships and lack the facility for displaying secondary variables.

**Results:**

Here we present Krona, a new visualization tool that allows intuitive exploration of relative abundances and confidences within the complex hierarchies of metagenomic classifications. Krona combines a variant of radial, space-filling displays with parametric coloring and interactive polar-coordinate zooming. The HTML5 and JavaScript implementation enables fully interactive charts that can be explored with any modern Web browser, without the need for installed software or plug-ins. This Web-based architecture also allows each chart to be an independent document, making them easy to share via e-mail or post to a standard Web server. To illustrate Krona's utility, we describe its application to various metagenomic data sets and its compatibility with popular metagenomic analysis tools.

**Conclusions:**

Krona is both a powerful metagenomic visualization tool and a demonstration of the potential of HTML5 for highly accessible bioinformatic visualizations. Its rich and interactive displays facilitate more informed interpretations of metagenomic analyses, while its implementation as a browser-based application makes it extremely portable and easily adopted into existing analysis packages. Both the Krona rendering code and conversion tools are freely available under a BSD open-source license, and available from: http://krona.sourceforge.net.

## Background

Metagenomics is a relatively new branch of science and much of the current research is exploratory. Visualization has thus been a prominent aspect of the field, beginning with the analysis package MEGAN [1,2]. Distilling metagenomic data into graphical representations, however, is not a trivial task. The foundation of most metagenomic studies is the assignment of observed nucleic acids to taxonomic or functional hierarchies. The various levels of granularity (e.g. ranks) inherent in these classifications pose a challenge for visualization. Node-link diagrams can be used to convey hierarchy, and bar or pie charts can relate abundances at specific levels, but neither of these methods alone creates a complete illustration of classificatory analysis. Furthermore, taxonomic and functional hierarchies are often too complex for all nodes to be shown, and wide variations in abundances can be difficult to represent. MEGAN addresses these problems by augmenting node-link diagrams with small, log-scaled quantitative charts at the nodes. This type of display is also used by the web-based metagenomic platform MG-RAST [3]. The approach has the advantage that nodes are explicitly represented in the hierarchy, regardless of magnitude. Its drawback, though, is that its disparate quantitative charts and logarithmic scaling obfuscate relative differences in abundances. Another web-based platform, METAREP [4], features naturally scaled heatmaps of abundance, but only for specific ranks. Both MG-RAST and METAREP can also display the relative abundances of children for individual nodes while browsing their hierarchies. A common strength of MEGAN, MG-RAST, and METAREP is that they facilitate direct comparison of multiple datasets at each node, such as metagenomes sampled from different regions or under different conditions. It is important to note, however, that these comparisons will be of predictions, rather than true abundances.

Metagenomic classification algorithms are constantly improving, but their results still come with a significant degree of uncertainty. Only a small fraction of the tree of life is represented in reference databases, and this causes widespread bias in classifications [5]. Uncertainty increases for more specific classifications, but can also vary widely among hierarchical branches. Thus, in order to properly interpret classificatory results, it is important to be able to make direct comparisons across multiple ranks simultaneously. This task is difficult or impossible with available visualizations. Moreover, most classification methods provide valuable information about the confidence of their predictions. This can be explicit, as the confidence estimates provided by the Ribosomal Database Project (RDP) Classifier [6] and PhymmBL [7,8], or inferred, as from the e-values of BLAST results [9,10]. Even though this information should be considered before drawing comparative conclusions, none of the tools discussed here provides a way of visualizing it with abundance. Radial space-filling (RSF) displays [11-15], however, allow both comparisons across multiple ranks and custom coloring, making them an attractive alternative to the typical visualizations. Hybrids of traditional pie charts and contemporary TreeMaps [16], these displays convey hierarchy implicitly via angular subdivision. As in TreeMaps, nesting lower levels within higher ones makes efficient use of space. However, since angular space increases with distance from the center, deeper levels of the hierarchy can be labeled without distortion. This property also creates a problem for metagenomics, though - the angular aspect diminishes for deep, broad hierarchies, making RSF displays infeasible for typical metagenomic taxonomies. To address the demands of metagenomic visualization, we have extended the capability of RSF displays with a novel layout algorithm, a polar-coordinate zooming technique, and rich interactive features. Additionally, to maximize portability and keep pace with the rapidly advancing field of metagenomics, we have implemented our method, entitled Krona, utilizing the emerging HTML5 standard. This allows interactive Krona charts to be shared via the Web and allows the Krona platform to be easily adapted into existing analysis frameworks. Finally, because metagenomic analysis tools continue to be introduced and refined, Krona is designed to be independent of these methods and flexible enough to be adapted to new ones.

## Implementation

### Architecture

**Figure 1:**
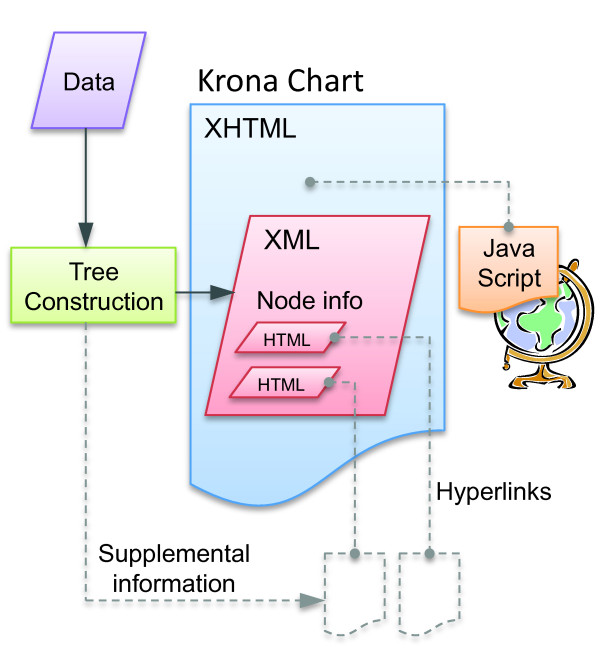
**The Krona architecture**. XML within an XHTML document is used to store chart data within a web page. XML tag nesting is used to describe the hierarchy, while attributes are used to store magnitude and other information about each node. Krona displays these attributes as HTML elements, allowing hyperlinks to supplemental pages for each node. These could be either pages created with the Krona chart, such as BLAST results, or existing web pages, such as NCBI taxonomy pages for the taxonomy IDs of the nodes. The Krona interface JavaScript is linked into the chart either via the Web or locally.

Thanks to technologies such as HTML5 and JavaScript, modern Web browsers are capable of rendering fully featured, graphical user interfaces for both Web sites and local applications. Krona's architecture takes a hybrid approach in which data are stored locally, but the interface code is hosted on the Internet. This allows each Krona chart to be contained in a single file, making them easy to view, share, and integrate with existing websites. The only requirements for viewing are an Internet connection and a recent version of any major web browser (though local charts that do not require an Internet connection can also be created and viewed with a Krona installation). Modularity is achieved by embedding XML chart data in an XHTML document that links to an external JavaScript implementation of the interface (Figure 1). When a web browser renders the XHTML document, the JavaScript loads chart data from the embedded XML and renders the chart to an HTML5 *canvas *tag. Hosting the JavaScript on the Internet avoids installation requirements and allows seamless, automatic updating as Krona evolves. To allow Krona to be used for a wide variety of applications, utilities for creating Krona charts are separated from the viewing engine. A package of these, called KronaTools, comprises Perl scripts for importing data from several popular bioinformatics tools and generic file types.

Hierarchical classifications can be directly imported from the RDP Classifier, Phymm/PhymmBL, MG-RAST (both taxonomic and functional), or the web-based bioinformatics platform Galaxy [17]. Sequences can also be taxonomically classified from BLAST results downloaded from NCBI [9,10] or the METAREP metagenomic repository [4]. Classification of raw BLAST results is performed by finding the lowest common ancestor of the highest scoring alignments (an approach similar to that of MEGAN), and data are mapped to a taxonomy tree automatically downloaded and indexed from the NCBI taxonomy database [18]. When importing classifications from RDP and PhymmBL a color gradient can be used to represent the average reported confidence of assignments to each node. For MG-RAST, METAREP, and raw BLAST results, the nodes can be colored by average log of e-value or average percent identity. Also, since Phymm/PhymmBL and BLAST classifications can be performed either on reads or assembled contigs, the scripts for importing from these tools allow the optional specification of magnitudes for each classified sequence. A script is also provided to generate magnitudes based on reads per contig from assemblies in the common ACE file format. Other types of classifications can be imported from basic text files or an Excel template detailing lineage and magnitude. Finally, an XML file can be imported to gain complete control over the chart, including custom attributes and colors for each node. Since node attributes can contain HTML and hyperlinks, XML import allows Krona to be deployed as a custom data browsing and extraction platform in addition to a visualization tool.

### Visual design

**Figure 2:**
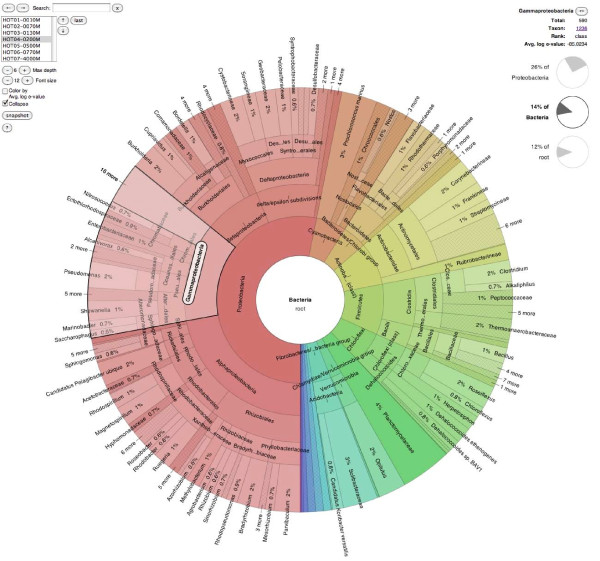
**The Krona RSF display**. The bacterioplankton metagenome from a vertical profiling of the North Pacific Subtropical Gyre [19] was imported from METAREP and displayed using Krona. Taxonomy nodes are shown as nested sectors arranged from the top level of the hierarchy at the center and progressing outward. Navigational controls are at the top left, and details of the selected node are at the top right. The chart is zoomed to place the domain "Bacteria" at the root and the taxon "Gammaproteobacteria" is shown selected. An interactive version of this chart is available on the Krona website.

The Krona display resembles a pie chart, in that it subdivides separate classes into sectors, but with an embedded hierarchy. Each sector is overlaid with smaller sectors representing its children, which are squeezed toward the outside of the chart to give the parent room for labeling. This does not cause distortion because, as in a pie chart, magnitudes are represented by the angle of each sector rather than the area. For example, Figure 2 shows an oceanic metagenome [19] imported from METAREP. The taxon "Gammaproteobacteria" is selected, and the angle of the highlighted sector indicates the relative magnitude of the node (in this case 110,467 classified sequencing reads, as shown in the upper right corner). The sector also surrounds smaller sectors, which represent constituents of Gammaproteobacteria. In this case, the sum of the constituent angles equals the angle of the parent, indicating that no assignments were made directly to Gammaproteobacteria. If assignments had been made to this internal node, its angular sweep would be wider than the sum of its children's, clearly showing both the summary and the assigned amount in relation to each other.

A common criticism of RSF displays is the difficulty of comparing similarly sized nodes. To make comparisons easier, Krona sorts nodes by decreasing magnitude with respect to their siblings. In addition, the nodes can be colored using a novel algorithm that works with the sorting to visually emphasize both hierarchy and quantity. This algorithm, which is enabled by default, uses the hue-saturation-lightness (HSL) color model to allow procedural coloring that can adapt to different datasets. First, the hue spectrum is divided among the immediate children of the current root node. Each of these children in turn subdivides its hue range among its children using their magnitudes as weights. Coloring each sorted node by the minimum of its hue range causes recursive inheritance of node hue by the largest child of each generation. The result is visual consistency for lineages that are quantitatively skewed toward particular branches. To distinguish each generation without disrupting this consistency, the lightness aspect of the HSL model is increased with relative hierarchical depth, with saturation remaining constant.

### Spatial efficiency

Metagenomic hierarchies can easily become too complex for all nodes to be discernibly apportioned and labeled on a computer screen. Although Krona ameliorates this problem with interactive zooming, it also offers several modifications to RSF displays that maximize the amount of information contained in each view.

First, radix-tree compression is used to collapse linear subgraphs in the hierarchy, simplifying the chart without removing quantitative relationships. Linear subgraphs, which represent multiple ranks of the same classification, occur when taxonomic classifications for a sample are mapped onto a full taxonomy tree. For example, if *Homo sapiens *were the only representative species of the class *Mammalia*, it would typically be redundantly classified under *Primates, Hominids*, and other ranks. To allow such classifications to be viewed, collapsing can be dynamically toggled, with animation depicting the transition. For additional simplification of complex trees, the taxonomy can be pruned to summarize the data at a specified depth. Figure 2, for example, shows an NCBI taxonomy summarized at a maximum depth of 6 levels and with linear subgraphs collapsed.

Second, since deeper taxonomical levels are often the most interesting (e.g. genus and species classifications), Krona allows significant quantities at these levels to be viewed in direct relation to the root of the hierarchy. This is accomplished by dynamically reducing the labeling area of intermediate classifications, removing their labels if necessary. Compression is increased moving outward from the center to ensure that the highest levels of the current view can also be labeled. The intermediate levels that have been compressed can always be seen more clearly by zooming.

Finally, Krona's labeling algorithms greatly increase textual information density compared to other RSF implementations. Space is used efficiently by orienting leaf node labels along radii and internal node labels along tangents. Internal labels use step-wise positioning and collision-based shortening to display as much text as possible while avoiding overlaps.

### Polar-coordinate zooming

Because radial space-filling displays recursively subdivide angles, the shapes of the nodes approach rectangles as hierarchical depth increases and as node magnitudes decrease. Thus, zooming small nodes by simply scaling the entire figure in Cartesian coordinate space would result in a loss of the angular aspect that makes RSF displays intuitive and space-efficient. To increase the capacity of the displays without causing this problem, Krona uses a polar coordinate space for zooming. This is accomplished by increasing the angular sweep and radius of the zooming node until it occupies the same circle as the original overview. The angular sweeps of surrounding nodes are decreased simultaneously, creating an animated "fisheye" effect. This animation ensures user cognition of the change in context, and the final zoomed view retains the entire capacity as the original. Zooming can then be repeated for any node with children, providing informative views of even the deepest levels of a complex hierarchy. Zooming out to traverse up the hierarchy can be accomplished similarly by clicking ancestral nodes, which are shown in the center of the plot and as summary pie charts next to the plot. This triggers the reverse of the fisheye animation, compressing the current node to reveal its position in the new, broader context.

### Multi-dimensional data

**Figure 3:**
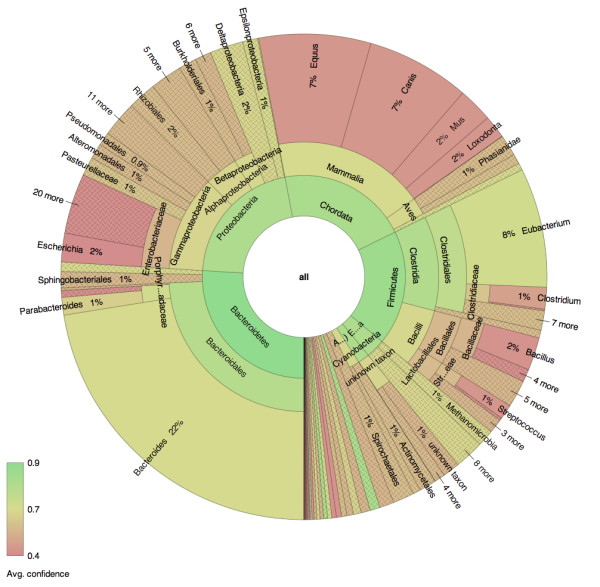
**Coloring by classification confidence**. Human gut sample MH0072 from the MetaHIT project [23] was classified using PhymmBL and displayed using Krona. Abundance can be simultaneously visualized with an accessory attribute by linking it to hue. In this example, hue is used to display classification confidence as reported by PhymmBL. The average confidence value for each node is colored from low (red) to high (green), distinguishing uncertain from certain classifications. An interactive version of this chart is available on the Krona website.

To visualize secondary attributes in addition to magnitude, individual nodes in Krona may be colored by variable. For categorical variables, users may define the color of every node in the XML. For quantitative variables, a gradient may be defined that will color each node by value. An example of this is shown in Figure 3, where each node is colored by a quantitative red-green gradient representing classification confidence.

Additionally, metagenomic data are often generated at discrete points across multiple locations or times. Krona is able to store the data from multiple samples in a single document. Individual samples may then be stepped through, at any zoom level, using the navigation interface at the top left. For example, in Figure 2 Krona is displaying one of seven depth samples from the oceanic water column. Advancing through these samples progresses through samples at greater and greater depths. The transition between samples is animated using a polar "tween" effect, emphasizing the difference between samples. The result of this style of navigation is a series of moving pictures, where the taxa dynamically grow and shrink from sample to sample-in this case as sampling descends the water column. This approach is eye-catching for a few samples, but direct comparison between many samples simultaneously is difficult with radial charts. Analysis across many samples is better left to traditional heatmap and differential barchart visualizations.

## Results

### Interactive design

The design of Krona addresses the seven key tasks recommended for productive interactions with dynamic visualizations [20]: *Overview*, *Zoom*, *Filter*, *Details-on-demand*, *Relate*, *History*, and *Extract*. The initial radial space-filling display showing the first several ranks of the hierarchy serves as an *overview*, while *relation *of quantitative and hierarchical properties of the nodes is conveyed by angular sweep and optionally by color. Any visible node can be selected to reveal *details*, which can include attributes, descriptions, and HTML elements, including hyperlinks. The selected node can then be *zoomed *so it fills the view, revealing its sub-hierarchy in more detail. To reduce clutter, a semantic zoom joins small, adjacent nodes into groups to create easily discernable regions, and to provide an overview while zoomed, ancillary charts display the position of the current view relative to higher levels. These can also be selected to zoom out from the current view, refocusing at the selected level. A *history *of zooming actions is also kept to allow users to retrace their traversal of the hierarchy. If multiple datasets are present in a chart, the view can be switched between them while at any zoom level, showing an animated transition to remain oriented. To *filter *the chart by node names, a textual search function highlights both matching nodes and nodes that contain hidden matches. Finally, at any point while exploring data with Krona, users may *extract *their favorite figures as publication-ready SVG files for later reference.

### Evaluation

**Figure 4:**
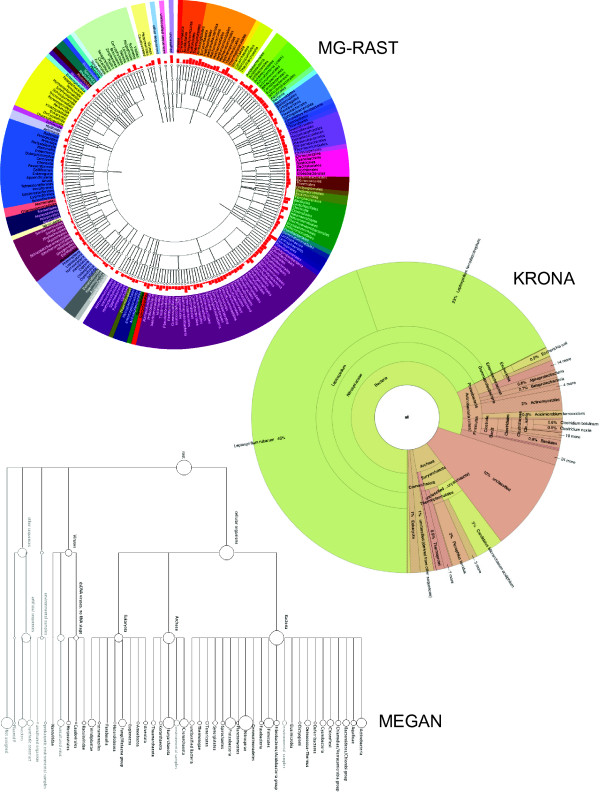
**Comparison of three hierarchical display strategies**. The acid mine drainage metagenome [21] was classified using MG-RAST and displayed using MG-RAST, MEGAN, and Krona. MG-RAST and MEGAN augment hierarchical node-link diagrams with log-scaled, quantitative charts. Krona displays abundance and hierarchy simultaneously using a radial space-filling display. The Krona chart features a red-green color gradient signifying average e-values of BLAST hits within each taxon, with red being the highest observed e-value (least significant) and green being the lowest (most significant). An interactive version of this chart and a second chart displaying the functional classifications of the same dataset are available on the Krona website.

An effective visualization should display the data in such a way that the answers to common questions are obvious. For metagenomics, Krona aims to answer questions regarding the relative abundance of taxa across multiple levels of the hierarchy simultaneously. To evaluate Krona's utility for metagenomics, we chose to compare it against two other commonly used metagenomic visualizations from the MG-RAST and MEGAN toolkits. All three programs were used to visualize the famous metagenome of an acid mine drainage biofilm [21]. MG-RAST was used to create taxonomical and functional classifications of sequencing reads from the sample. Figure 4 shows the taxonomical classification of the same sample viewed with MG-RAST, Krona, and MEGAN. The three charts have been limited to the same physical dimensions to simulate typical screen or document space, with the direct comparison highlighting the relative strengths of Krona. First, because of its naturally scaled, space-filling display, Krona is able to display information at all levels of the hierarchy for the most abundant taxa, from the domain to the species rank. MEGAN and MG-RAST, however, utilize a fixed-width layout that forces them to summarize abundance at higher ranks (phylum and order) to fit in the same space, limiting the scope of their overview.

For example, the question "What are the most abundant domain and species?" can be easily answered by the Krona plot as "Bacteria" and "*Leptospirillum rubarum*". In comparison, the name of the most abundant domain is not available with MG-RAST, which only displays labels and abundance at the leaves and does not summarize internal nodes like Krona and MEGAN. The MEGAN plot does show bacteria as the most abundant domain, but its log scale suggests that bacteria and archaea are similarly abundant. The Krona plot, however, shows the true abundance estimates of bacterial and archaean sequences are roughly 80% and 10% of the sample, respectively. As for the most abundant species, only Krona displays enough ranks of the hierarchy to make this clear at first glance. Even at the higher ranks, comparisons of abundance are difficult in the MG-RAST and MEGAN displays due to the small, log-scaled charts. Evaluation of lower levels in these tools requires expanding the trees beyond the available screen space, which further hinders comparison. In cases where the rare components of a metagenome are of interest, the fixed-width displays of MG-RAST and MEGAN may be justified. However, Krona's interactive search and polar zoom supports drill-down to even the smallest magnitudes, following the information seeking mantra of overview first to guide discovery, then zoom and filter [20]. Furthermore, Krona's coloring of the chart by e-value makes it clear that the BLAST analysis can differentiate the two most abundant species of Leptospirillum with relatively high confidence, while many other abundance estimates are less confident even at the phylum level. The presence of these two species is consistent with the characterization of this metagenome, but would not be immediately evident with other visualizations.

The acid mine drainage dataset discussed above is a relatively "simple" metagenome. Figure 2 shows a much more complex metagenomic profile of bacterioplankton in the North Pacific Subtropical Gyre water column. This dataset was imported into Krona from METAREP, which hosts BLAST results for sequencing reads, and comprises seven separate samples at depths ranging from 10 meters to 4000 meters. The multiple samples can be browsed using the navigational controls, as described previously in the Implementation section. In addition, each taxon is annotated with the total number of fragments assigned, the average log-scaled e-value of the corresponding BLAST hits, and the GenBank taxon ID with HTML link-out. Figure 2 displays only the bacterial component at a static depth of 200 meters and summarized at a maximum hierarchical depth of six levels. To experience the interaction provided by the Krona interface, readers are encouraged to explore the dynamic version of this chart, available from the Krona website [22].

Figure 3 shows sequencing reads from a human gut sample (MH0072) from the MetaHIT project [23] as classified by PhymmBL 3. As in Figure 4, a red-green color gradient is used to convey confidence from low to high. However, PhymmBL 3 provides normalized confidence values for each rank (from phylum to species) of each classification. This chart clearly shows that classification confidence decreases for deeper levels of the taxonomy, and that Bacteroides and Eubacterium are both highly abundant and relatively confident classifications in comparison to the other taxa. Also in this dataset, it is evident that there remains non-specific mammalian DNA (after subtraction of human specific reads). Classification at the mammalian level is confident, but the DNA sequence is either highly degraded or the source is not in the reference database. This is another example of how Krona's coloring and hierarchical relationships can reveal trends in classification data that other visualizations would not.

## Conclusions

Krona supplements existing metagenomic visualizations by creating clearer depictions of abundance estimates and by enabling in-depth understanding of the underlying classifications. It leverages recent advancements in the field of information visualization and introduces new methods of interaction. Moreover, it is not tied to a specific analysis toolkit and is designed to be a generic and modular visualization, capable of benefiting a wide variety of applications within and beyond metagenomics. Consequently, much of Krona's strength comes from its lightweight implementation and its ease of integration into existing and powerful Web analysis portals such as MG-RAST, METAREP, and Galaxy. Furthermore, to the best of our knowledge, Krona is the first bioinformatics tool built completely on HTML5 and serves as a demonstration of the power of emerging Web technologies for creating widely applicable and highly accessible visualization tools.

## Availability and requirements

**• Project name: **Krona

**• Project home page: **http://krona.sourceforge.net

**• Operating system: **Platform independent

**• Programming language: **HTML5, JavaScript, Perl

**• Other requirements: **Chrome 7.0+ (recommended for performance), Safari 4.0+, Firefox 3.5+, Opera 10.5+, or Internet Explorer 9.0+

**• License: **BSD

## List of abbreviations

HTML5: HyperText Markup Language, version 5; RSF: Radial Space-Filling; SVG: Scalable Vector Graphics; XHTML: eXtensible HyperText Markup Language; XML: eXtensible Markup Language.

## Authors' contributions

BDO conceived, designed and programmed the software and drafted the manuscript. NHB contributed to the design of the software and generated test data. AMP contributed to the design of the software and drafted the manuscript. All authors read and approved the final manuscript.
